# The *Botrytis cinerea* Gene Expression Browser

**DOI:** 10.3390/jof9010084

**Published:** 2023-01-06

**Authors:** Gabriel Pérez-Lara, Tomás C. Moyano, Andrea Vega, Luis F. Larrondo, Rubén Polanco, José M. Álvarez, Daniel Aguayo, Paulo Canessa

**Affiliations:** 1 Centro de Biotecnologia Vegetal, Facultad de Ciencias de la Vida, Universidad Andres Bello, Santiago 8370186, Chile; 2 ANID–Millennium Science Initiative–Millennium Institute for Integrative Biology (iBIO), Santiago 7500565, Chile; 3 Facultad de Ingenieria y Ciencias, Universidad Adolfo Ibañez, Santiago 7941169, Chile; 4 ANID–Millennium Science Initiative–Millennium Nucleus for the Development of Super Adaptable Plants (MN-SAP), Santiago 8331150, Chile; 5 Center of Applied Ecology and Sustainability (CAPES), Santiago 8331150, Chile; 6 Departamento de Genetica Molecular y Microbiologia, Facultad de Ciencias Biologicas, Pontificia Universidad Catolica de Chile, Santiago 8331150, Chile; 7 Center for Bioinformatics and Integrative Biology, Facultad de Ciencias de la Vida, Universidad Andres Bello, Santiago 8370186, Chile; 8 Agricultura Digital, Servicio Agrícola, Salinas y Fabres S.A., Ruta 5 Sur, Parcela 165, Hijuela Larga, Paine 9540000, Chile

**Keywords:** *Botrytis cinerea*, RNA-Seq, phytopathogen, transcriptomics, *Botrytis* Expression Browser, BEB

## Abstract

For comprehensive gene expression analyses of the phytopathogenic fungus *Botrytis cinerea*, which infects a number of plant taxa and is a cause of substantial agricultural losses worldwide, we developed BEB, a web-based *B. cinerea* gene Expression Browser. This computationally inexpensive web-based application and its associated database contain manually curated RNA-Seq data for *B. cinerea*. BEB enables expression analyses of genes of interest under different culture conditions by providing publication-ready heatmaps depicting transcript levels, without requiring advanced computational skills. BEB also provides details of each experiment and user-defined gene expression clustering and visualization options. If needed, tables of gene expression values can be downloaded for further exploration, including, for instance, the determination of differentially expressed genes. The BEB implementation is based on open-source computational technologies that can be deployed for other organisms. In this case, the new implementation will be limited only by the number of transcriptomic experiments that are incorporated into the platform. To demonstrate the usability and value of BEB, we analyzed gene expression patterns across different conditions, with a focus on secondary metabolite gene clusters, chromosome-wide gene expression, previously described virulence factors, and reference genes, providing the first comprehensive expression overview of these groups of genes in this relevant fungal phytopathogen. We expect this tool to be broadly useful in *B. cinerea* research, providing a basis for comparative transcriptomics and candidate gene identification for functional assays.

## 1. Introduction

The collection of genome-scale gene expression data by RNA sequencing (RNA-Seq) is an important strategy in modern molecular biology. Biological models across phyla have benefited from recent technological advances in massive sequencing methodologies, including short and emerging long-read transcriptomics [1]. Accordingly, accurate gene expression profiling can be achieved in virtually any organism and experimental condition. Thus, RNA-Seq represents a tool that can provide clues regarding the function and regulation of diverse genes of interest [2].

The standard workflow for RNA-Seq experiments relies on high-quality RNA extraction. After ensuring an adequate quantity and quality of nucleic acids [3], sequencing libraries are built following rigorous and standardized methods. Low-quality reads and adapter sequences must be discarded before differential gene expression analyses [4]. These data provide a basis for time-consuming mapping of each sequence read to a reference genome using specialized short-read alignments tools, such as STAR [5], TopHat2 [6], Hisat2 [7], or Kallisto [8], for quantification [9,10] and ultimately for differential expression analyses [11,12]. Readers can consult several reviews to address critical considerations at each step [1,13,14,15]. After these massive scientific and computational efforts to make RNA-Seq data biologically accurate, meaningful, and accessible to most biologists, RAW sequence files are deposited into public databases, such as the NCBI Sequence Read Archive (SRA) [16]. Therefore, there are significant scientific, technical, and computational challenges, particularly when bioinformatics expertise or computational power are lacking, in the analysis of cross-laboratory experiments to determine otherwise hidden global gene expression patterns. 

Several initiatives—most aimed at well-known model species—have been propelled to circumvent some of the abovementioned difficulties. For example, data for the model plant species *Arabidopsis thaliana* [17], agriculturally relevant plants including tomato, rice, wheat, maize, and barley [18], and several other species have been aggregated in massive initiatives, such as the “Expression Atlas” of the European Bioinformatics Institute (EMBL-EBI) [19]. This latter collection holds information on 22 animal models, over 9 plant species other than *Arabidopsis*, and only 3 fungal species (*Aspergillus fumigatus*, *Schizosaccharomyces pombe* and *Saccharomyces cerevisiae*), with 95.7% of fungal RNA-Seq experiments concentrated on the baker’s yeast. Therefore, tools and curated gene expression databases for additional fungal species would provide an opportunity to better understand the biology of this relevant but often neglected group of organisms [20]. 

One notable exception is the wheat fungal pathogen *Puccinia striiformis* f. sp. *tritici*, with a recently developed platform for analyzing gene expression patterns in a myriad of culture conditions, including “*in planta*” growth [21]. This strategy can provide meaningful insights regarding, for instance, genes involved in plant infection. While fungi represent an exceptional biotechnological chassis, their extraordinary capacity for adaptation to diverse environmental niches also presents a risk for animal health and agricultural production [20,22]. Despite the availability of a relatively small but significant number of transcriptomics experiments for most relevant fungal phytopathogens (Table 1), a simple and easy-to-use tool to evaluate gene expression patterns is lacking. As an important specialist phytopathogenic fungus, *Magnaporthe oryzae* has a high degree of host specificity [23] and is the causal agent of rice blast disease. In contrast, the most relevant generalist is the so-called gray mold fungus *Botrytis cinerea* [24]. Both have an enormous negative impact on food security and production worldwide. 

*B. cinerea* is the most extensively investigated necrotrophic fungal plant pathogen, with a history of research spanning several decades. This single fungal species explains over $10 billion in agricultural product losses [25]. In the genus *Botrytis*, several species are specialist plant pathogens [26]. In contrast, with its necrotrophic infection strategy, *B. cinerea* can infect over 1000 plant species [27]. For interested readers, there are several seminal works revisiting canonical *B. cinerea* infection strategies [28,29,30]. Contemporary research trends and recent advances have been reviewed elsewhere [27,31,32,33,34,35,36]. 

Since the foundational analyses that contributed to the first genome database for *B. cinerea* [37], several assemblies improvements have been made [38]. This led to a gapless genome whose assembly was supported by an optical map [39]. These genomic advances have provided a basis for a significant number of transcriptomic experiments (Table 1), which remain largely underexplored owing to the lack of tools to analyze all expression data from multiple sources simultaneously.

To visualize organism-wide gene expression patterns in *B. cinerea*, we developed the *B. cinerea* gene Expression Browser (BEB). With a user-friendly interface, this tool and database (available at http://beb.canessalab.org, accessed on 30 November 2021) allow straightforward expression analyses of genes of interest under various conditions. For this purpose, users only need to provide *B. cinerea* gene IDs. To demonstrate the usability and effectiveness of this tool, we analyzed several genes of interest, including virulence factors. Among all genes encoding NRPS (non-ribosomal peptide synthetases) and PKS–NRPS (polyketide synthetases–NRPS hybrids) in *B. cinerea*, we discovered that two genes (*bcnrps7* and *bcpks5*) display remarkably similar expression patterns across all experimental conditions. Highlighting the utility of BEB, the observed expression pattern supports the role of the uncharacterized *bcpks5* in siderophore biosynthesis, a relevant iron acquisition mechanism in fungi [40,41]. Finally, taking advantage of BEB as a curated gene expression database, we propose a set of new reference genes that can be used in RT-qPCR studies.

## 2. Materials and Methods

### 2.1. RNA-Seq Datasets Available for Botrytis cinerea

To generate a robust web-based platform capable of visualizing global gene expression patterns in *B. cinerea* across available experiments, all publicly available RNA-Seq data were retrieved from the NCBI Sequence Read Archive (SRA) as well as the EMBL—EBI (European Bioinformatics Institute), accessed 30 November 2021. The “*Botrytis cinerea*” search term was used to identify datasets. The downloaded dataset was composed of 218 individual files (including replicates) representing 76 experimental groups (or conditions). RNA-Seq experiments included, but were not limited to, those with *B. cinerea* growing in axenic *in vitro* cultures (non-infective conditions, i.e., on plate and liquid medium) and during the infection of different plant species (dual RNA-Seq; e.g., *B. cinerea* infecting *A. thaliana*, among others), relevant conditions for a phytopathogen such as *B. cinerea*. Details are provided in Supplementary Material 1.

### 2.2. Data Pre-Processing and RNA-Seq Experiment Mapping

Since several different RNA-Seq datasets were included in this study, adequate quality control of sequencing data is essential to determine accurate expression patterns. As RNA-Seq data were based on both single-end (SE) and paired-end (PE) Illumina sequencing technologies, a careful examination of the data was performed before mapping. First, quality was assessed using fastQC (version 0.11.8, [42]). Then, low-quality reads and sequencing adapters from FASTQ files were filtered out using BBDuk (version 38.90, https://sourceforge.net/projects/bbmap/, accessed on 30 November 2021). Quality thresholds were as follows: ktrim = r k = 23 mink = 11 hdist = 2 qtrim = rl trimq = 10 ftm = 5 maq = 15 minlength = 50 tbo. Filtered reads were pseudoaligned to the *B. cinerea* B05.10 transcriptome [39] (ASM83294v1) using Kallisto (v0.46.0) [8]. The *B. cinerea* reference transcriptome was downloaded from EnsemblFungi release 52 [43], representing the previously published work [39]. Kallisto SE mapping was performed under the following settings: –single -b 100 -l 100 -s 20. Standard parameters (-b 100) were employed for PE mapping. As recently reported in an exhaustive benchmark evaluation [44], Kallisto is effective for large datasets, such as the one reported herein. In addition, since this software is accurate and fast during the mapping and quantification procedure, it will be useful for keeping BEB up-to-date as more RNA-Seq datasets become available in the future.

### 2.3. Gene Expression Metadata Construction

**Figure 1:**
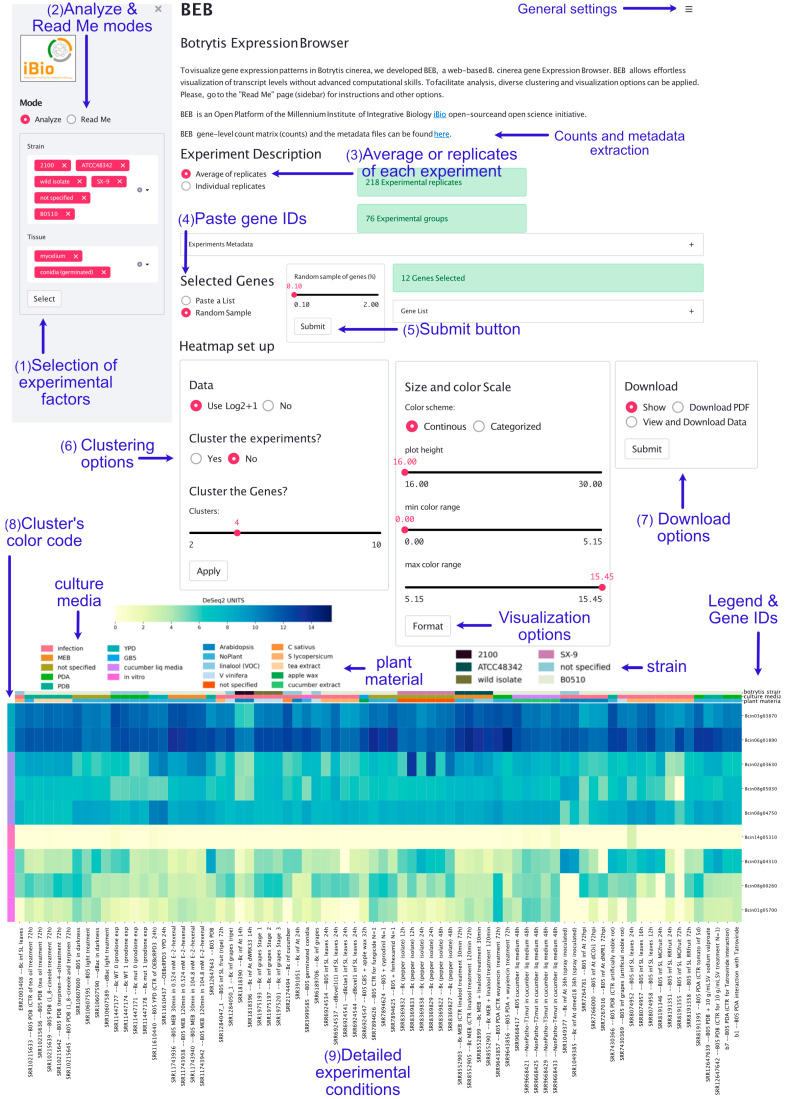
*B. cinerea* gene Expression Browser (BEB) graphical user interface. The BEB landing page contains a left sidebar section where experimental factors can be selected (1). The “Analyze” and “Read Me” modes (2) are available on the left sidebar (top section). Detailed instructions on how to use BEB are available in the latter display tool. In the upper section, users can choose to display average expression values or values for each replicate individually (3). Users can copy, paste, and submit gene IDs in the middle right section after selecting the “Paste a List” option (4). After clicking the “Submit” button (5), a heatmap depicting gene expression levels is generated at the bottom of the webpage. Clustering (6) and download options (7) are available in the middle section. BEB clusters are denoted with a color code shown at the outmost left column of the heatmap (8). A detailed description of each experiment is provided at the bottom of the figure, including SRA IDs (9).

The manually curated metadata file available on the BEB (Supplementary Material 1) uses the NCBI SRA metadata information schema [16] and describes the general experimental conditions of each RNA-Seq experiment. It is composed of eleven columns describing the following data: “experiment_ID” (SRA ID or identifier); “experiment_description” (manual annotation of experiments reported herein); “botrytis_strain,” “tissue,” “culture_media” (relevant information for *in vitro* cultures and *<<infection>>* experiments); “treatment,” “plant_material,” and “plant_tissue” (if available, describing the presence and type of plant material infected by the fungus); and “time.” In the tenth column of Supplementary Material 1, the metadata file also indicates the replicates of each experiment (described as “group_for_averaging”). This entry is used by BEB to generate a heatmap depicting gene expression values for each replicate or their average (see visualization options described in Figure 1). Importantly, not all RNA-Seq data available in the NCBI SRA contained a complete description of experimental conditions (e.g., information to cross-check the data with the associated sequencing file). Thus, when possible, associated publications were analyzed, making every effort to obtain as much information as possible. If available, PubMed IDs for the respective publication were also included (“PMID,” in the eleventh column in the metadata file). RNA-Seq experiments for which it was impossible to determine the FASTQ file confidently and respective experimental conditions were not included in the current implementation of BEB. Studies focused on small RNAs were omitted. Importantly, the file format used in Supplementary Material 1 is consistent with the CSV schema used by the BEB server (see instructions on the GitHub repository below). 

### 2.4. Gene Expression Analysis and the BEB Transcriptional Profile Database

Mapped read counts obtained using Kallisto were further processed to infer transcript abundances with the tximport package in R [45] (v1.20.0; on RStudio v4.1.0). This approach yielded a complete dataset containing gene-level counts derived from all RNA-Seq experiments. To determine gene expression levels in BEB, a custom Python version of the DESeq2 median of ratios method for normalization was used [11,46]. Detailed information on its implementation can be found in a GitHub repository (https://github.com/bioquimico/biber/tree/main/biber, accessed on 30 November 2021). To visualize gene expression levels, the gene-level count matrix and the metadata file described above are available on the BEB (see below).

### 2.5. BEB Server Implementation

To build an interactive browser to determine gene expression patterns in *B. cinerea*, we took advantage of various open-source computational technologies. The BEB server uses Streamlit’s open-source app framework (https://github.com/streamlit/streamlit, accessed on 30 November 2021) and Docker [47]. The latter allows the straightforward implementation of a local copy of the tool (e.g., to analyze in-house built RNA-Seq datasets) or, alternatively, to run a copy of the BEB on a different (dedicated) server employing a custom-built database for any organism of interest. All details are described on GitHub (https://github.com/bioquimico/biber/tree/main/biber, accessed on 30 November 2021). The code for the BEB was written in Python 3.7 using libraries that include NumPy [48], SciPy [49], Pandas [50], and Matplotlib [51], among others. The SciPy “maxclust” option was used for the clustering analysis (average distance). The metadata file and gene-level count matrix mentioned above as well as the Python code for BEB were used to arrange the data, set up, and run the web-based tool presented herein. The detailed BEB data processing flow chart is provided in Figure S1. All details are described on the mentioned GitHub repository. A working version of BEB is available at http://beb.canessalab.org. 

### 2.6. Additional Bioinformatics Analyses

To predict secondary metabolite (SM) gene clusters in the *B. cinerea* genome, antiSMASH (version 6.1.1) [52] was used employing default parameters. FASTA and GFF3 files from the *B. cinerea* genome database were provided, and the output was manually inspected. For the genes on chromosomes 17 and 18 (see below), BLAST2GO [53] was used to retrieve all available functional annotations. Owing to the quantity of expression data deposited in BEB, putative/new reference genes for reverse transcription quantitative real-time PCR (RT-qPCR) studies were identified. For this purpose, read counts for each *B. cinerea* gene were normalized with the total mapped reads per library. An additional normalization was then performed by upper quartile and median normalization, as described previously for RNA-Seq data [54]. Finally, the normalized reads of each gene were standardized by the transcript size and classified by quartiles of coefficient of variation (CV). Candidate reference genes have the lowest CV, as demonstrated previously [54,55,56]. 

## 3. Results

### 3.1. A Glimpse into the B. cinerea Expression Browser Graphical User Interface

The BEB landing page contains a left sidebar where experimental factors—extracted from the experimental metadata file described in the Materials and Methods—can be selected through different dropdown lists (Figure 1). Only “Strains” and “Tissue” are displayed by default. To view additional factors (e.g., time), the “Select” button (available at the bottom of the section) must be pressed. The “Read Me” option allows users to access detailed instructions, including visualization options and other more complex tasks (e.g., database update).

By default, in the middle section of the landing page, a list of randomly selected genes is used as an entry to generate a heatmap depicting gene expression values at the bottom. A space-separated gene list can be submitted after selecting the “Paste a List” option in the middle right section. Importantly, gene identifiers must be separated by spaces (in the form of Bcin[XX]g[YYYYY], where “XX” corresponds to the chromosome and “YYYYY” to the gene number). Once visualization parameters are selected and submitted, the bottom section shows a customizable heatmap. This graphical representation depicts the expression levels of the provided subset of genes in the experiments that fulfill the selected experimental conditions (Figure 1). 

BEB visualization capabilities are robust and allow comparisons of several genes simultaneously. For example, genes and experimental conditions can be clustered for the identification of co-expressed genes and expression trends. The biological relevance of this visualization option is exemplified in different Figures (see below). The heatmap customization parameters include coloration of expression levels by quartiles (see below), DESeq2 units, or log2-transformed values to highlight fold differences among experiments. The exploratory capacity of BEB also facilitates parallel comparisons of large sets of genes, as exemplified in an analysis of 176 protein-encoding genes whose products were detected in proteomics studies (see below). To maintain the overall performance of BEB, the number of genes that can be visualized at once from all available experiments is limited to 250. In comparison, the *Arabidopsis* eFP browser [57] supports only 40 genes at a time. Being the first filamentous fungus to be sequenced, the *Neurospora crassa* tool embedded in fungiDB [58,59] only contains 20 RNA-Seq datasets and does not generate heatmaps. Previously established tools do not allow for the determination of differentially expressed genes between two specific conditions. As BEB also serves as a manually curated database of genome-wide expression data, this task can be achieved. For this purpose, pre-mapped and pre-quantified RNA-Seq reads in BEB (the gene-level count matrix described in the Materials and Methods) (see Figure 1) can be used as input in locally installed tools, such as RobinNA [60], or easy-to-use online tools, such as iDEP [61]. These read counts can be further examined by more complex bioinformatic analyses (exemplified in Figure S2). As more RNA-Seq datasets become available, this table (and associated metadata file) will be expanded in http://beb.canessalab.org, as detailed herein and in the online information. We will rely on Kallisto software for accurate mapping and rapid quantification of RNA-Seq data, as described previously [44].

### 3.2. Global Gene Expression Patterns of Phytotoxic Secondary Metabolite Gene Clusters in B. cinerea

**Figure 2:**
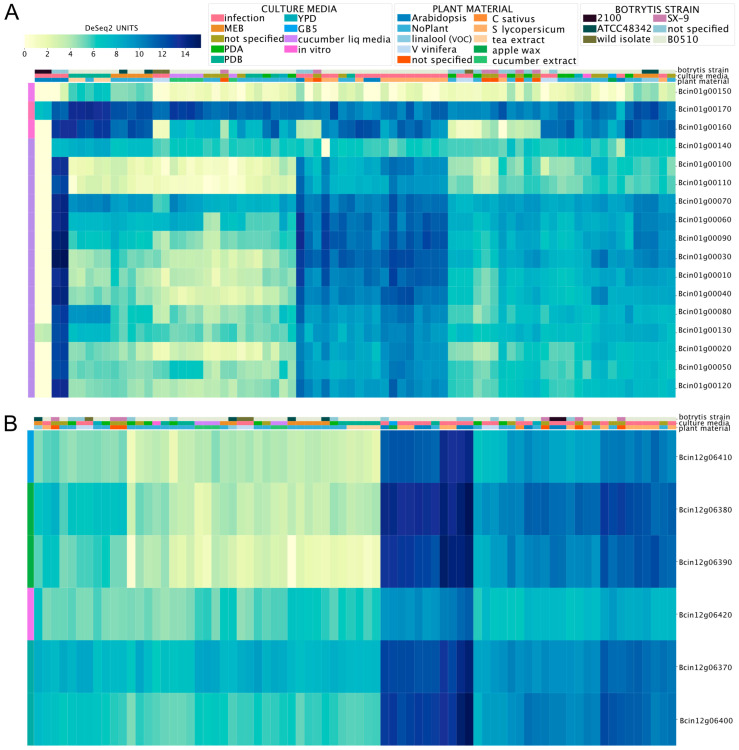
Expression patterns of gene clusters involved in the production of the phytotoxins botcinic acid (**A**) and botrydial (**B**) in *B. cinerea*. The heatmaps depict mRNA levels as calculated using DESeq2 (see Materials and Methods). The color scale represents the gene expression level from low to high (yellow to dark blue, respectively). The color code at the top of each heatmap indicates the culture media, plant material, and *B. cinerea* strain (key is shown above). Experimental conditions are indicated in each column, while genes are shown in each row. To streamline the overall figure, the description of each experiment was omitted from both heatmaps.

Biosynthetic gene clusters (BGCs) in *B. cinerea* are common. Since the first version of its genome project [37], at least 40 groups of genes that orchestrate the synthesis of secondary metabolites (SM) have been identified. One of these SMs is botcinic acid, a phytotoxic polyketide produced by the coordinated action of a gene cluster in a subtelomeric region of chromosome (Chr) 1 [62]. The transcription factor (TF) BcBoa13 (Supplementary Material 2) contributes to the transcriptional regulation of this cluster [63]. The BEB-generated heatmap plot for these genes (Supplementary Material 2, Figure 2A) shows low expression in PDA, PDB (Potato Dextrose Agar or Broth, respectively), YPD (Yeast Extract–Peptone–Dextrose), and MEB (malt extract broth) culture media *in vitro*. The highest values were detected during the infection of plants, including *Solanum lycopersicum* and *A. thaliana* (middle section of Figure 2A). This observation is consistent with those of previous studies showing that genes involved in botcinic acid synthesis are induced during the infection process [63]. The most notable exceptions were Bcin01g00150, Bcin01g00160, and Bcin01g00170 (depicted at the top of Figure 2A). These genes are physically located at one of the cluster’s borders.

Another relevant BGC in *B. cinerea* is required for botrydial production, an additional phytotoxic SM synthesized by this fungus. When explored on the BEB, the *bot* genes needed for botrydial synthesis show a similar expression pattern to that observed for genes involved in botcinic acid synthesis, with higher mRNA levels during the infection of plant tissues (see Figure 2B). The BEB clustering algorithm (see Materials and Methods) is denoted by a color code shown in the left-most column of the respective heatmap and facilitates the recognition of distinctive gene expression patterns under different conditions. It also allows for capturing distinct patterns within genes. For example, after visual inspection of Figure 2B, we observed that the TF BcBOT6 (Bcin12g06420), which is central for the biosynthesis of botrydial [64], displayed the most distinct expression pattern in comparison with the five non-regulatory genes encoded in the cluster.

### 3.3. Gene Expression of Orphan Secondary Metabolite Gene Clusters

**Figure 3:**
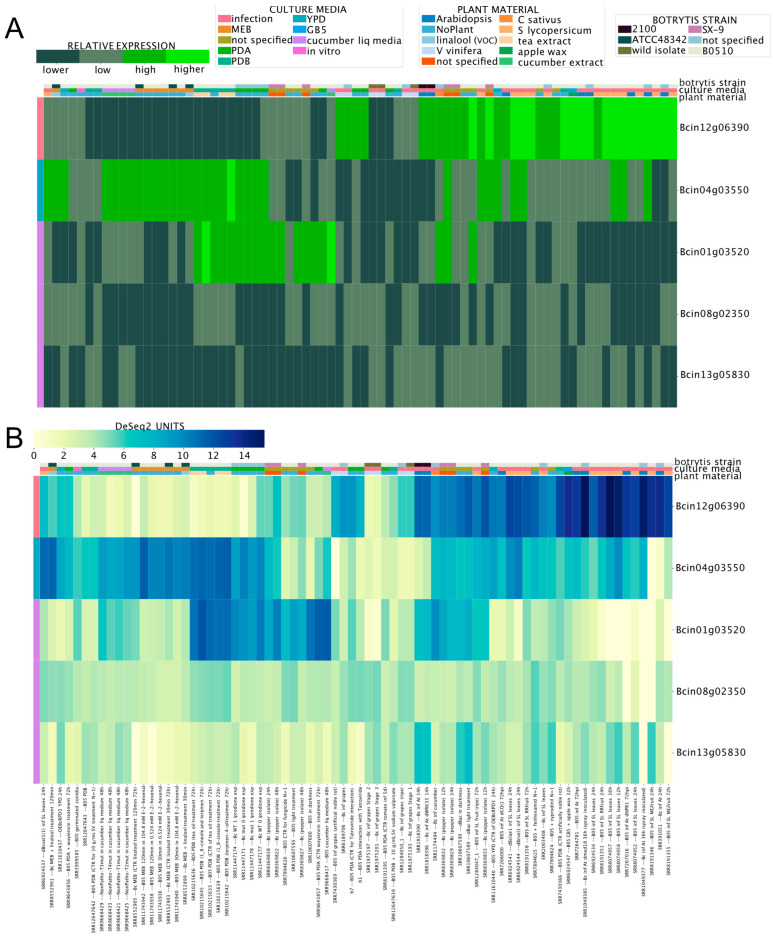
Expression patterns of five sesquiterpene cyclase enzyme-encoding genes. (**A**) Heatmap depicting the mRNA expression levels employing the BEB quartile-categorized expression option (lower: dark green; higher: light green). A continuous color scale (**B**) from low to high expression (yellow to dark blue, respectively) is also shown for comparative purposes. A color code at the top of each heatmap denotes culture conditions and *B. cinerea* strains. Experimental conditions and analyzed genes are indicated in columns and rows, respectively. Gene IDs are indicated at the right of each heatmap. A detailed description of each experiment is provided at the bottom of the figure, including SRA IDs for reference.

While several genes encoding enzymes required for SM synthesis have been identified in the genome of *B. cinerea* (Supplementary Material 3), many are predicted to participate in the synthesis of unknown compounds [65]. Since the expression patterns of these genes are unknown, we used the BEB to determine whether the newly developed tool can shed light on experimental conditions that could facilitate the study of SM biosynthesis. Among sesquiterpene cyclase-encoding genes, Bcin12g06390 (*bcbot2*; botrydial, see above; Supplementary Material 3) displayed the highest expression during the infection of *A. thaliana* and *S. lycopersicum*, which was clearly visualized using the BEB quartile-categorized expression option (Figure 3A). For comparative purposes, the continuous (color) scale is also displayed (Figure 3B). Most of the remaining genes showed relatively low expression values, with Bcin01g03520 and Bcin04g03550 as the most notable exceptions. Interestingly, the latter gene was highly expressed during the infection of *S. lycopersicum* and in cultures supplemented with cucumber or tea extracts.

**Figure 4:**
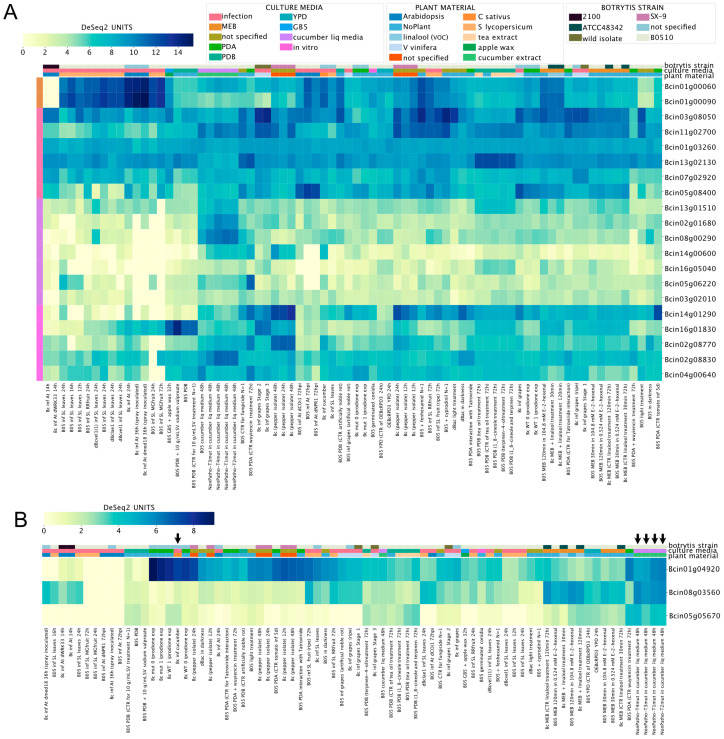
Polyketide synthetases (**A**, PKS) and diterpene cyclases (**B**) expression levels across experimental conditions. Both heatmaps indicate transcript levels with their corresponding expression scale from yellow to dark blue (low or high expression, respectively). The legend at the top of the figure denotes culture conditions and *B. cinerea* strains. Experimental conditions and genes are depicted in columns and rows, respectively. Gene IDs are provided at the right of each heatmap. A detailed description of each experiment is provided at the bottom of each heatmap (SRA IDs were not included). Columns denoted with arrows (in **B**) are discussed in the main text.

Among the polyketide synthetases genes (PKS, Supplementary Material 3), Bcin01g00060 and Bcin01g00090 (required for botcinic acid biosynthesis, see above) displayed the highest expression values during the infection of *A. thaliana* and *S. lycopersicum*, as shown in Figure 4A. In contrast, seven PKS genes (shown in the middle-bottom left of Figure 4A) exhibited low expression values in most culture conditions, with particularly low mRNA levels during the infection of tomato plants. Interestingly, among diterpene cyclases (Figure 4B, Supplementary Material 3), Bcin01g04920 showed the highest expression values during the infection of cucumber plants or in liquid media supplemented with cucumber extract (indicated by arrows in Figure 4B). These observations show how BEB can be used to generate new hypotheses, i.e., to identify experimental conditions for functional assays. 

**Figure 5:**
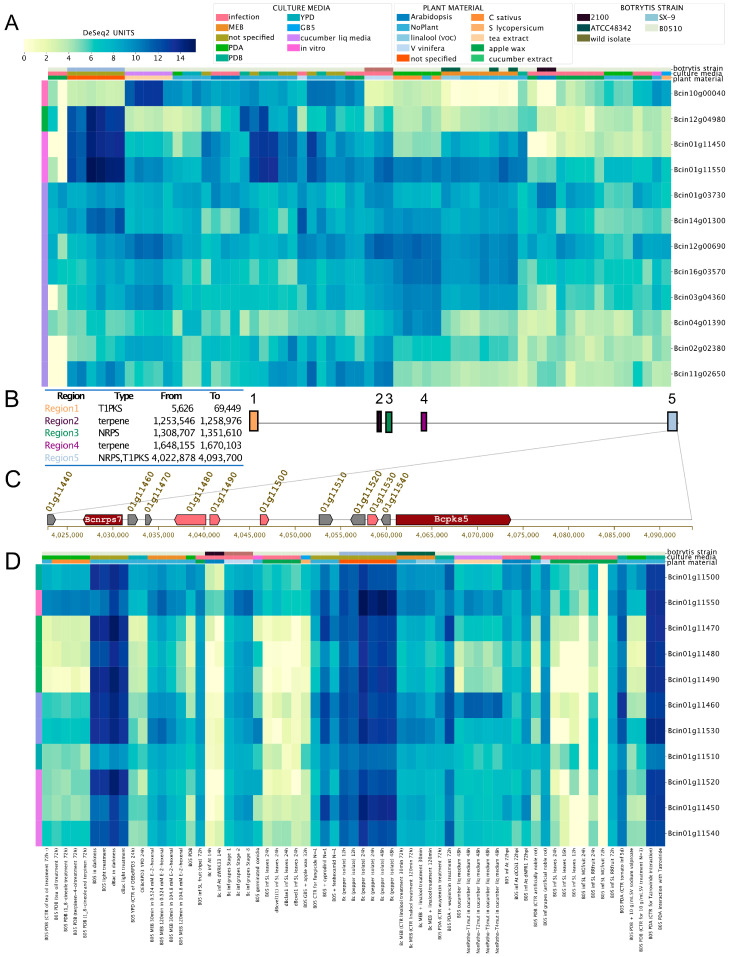
Expression patterns of genes encoding non-ribosomal peptide synthetases (NRPS) and hybrid polyketide synthetases (PKS-NRPS) in *B. cinerea*. (**A**) Expression patterns of all predicted NRPS and PKS-NRPS mentioned in Supplementary Material 3. To streamline the figure, the description of each experiment was omitted. (**B**) Identification of the five SM gene clusters on *B. cinerea* chromosome (Chr) 1. The table inset describes each of the five SM regions within Chr1, the predicted SM type, and their respective genomic coordinates. Colored boxes represent each region (right, with numbers). (**C**) Clustered genes within “Region 5” as described in (**B**). Boxes with arrowheads indicate each gene’s transcriptional orientation within the SM gene cluster. Dark red and pink arrowhead boxes indicate core and additional biosynthetic genes, respectively. Grey indicates other genes. Gene IDs and gene names are indicated. For simplicity, the “Bcin” prefix was omitted. (**D**) Expression patterns of all genes encoded in “Region 5” as indicated in (**B**,**C**), from *bcnrps7* to *bcpks5*. Both heatmaps indicate mRNA levels and experimental conditions as described in the former figures. In (D), a detailed description of each experiment is provided at the bottom of the heatmap (SRA IDs were not included).

Finally, we evaluated the expression levels of genes encoding NRPS (non-ribosomal peptide synthetases) and hybrid PKS–NRPS (Supplementary Material 3). Gene IDs Bcin01g11450 (putatively involved in ferrichrome siderophores biosynthesis) and Bcin01g11550 (unknown polyketide) showed very similar expression patterns, as indicated by the BEB clustering function (left-most column in Figure 5A). These two genes were clustered in a genomic region spanning *circa* 45 kbp depicted in Figure 5B,C (Region 5), a subtelomeric region in Chr 1 opposite to the botcinic acid SM cluster (Figure 5B, Region 1). The physical proximity of Bcin01g11450 and Bcin01g11550 and their particular expression patterns displayed in BEB heatmaps prompted us to investigate whether all genes within the 45 kbp region represent a putative BGC. According to antiSMASH [52], which allows the *in silico* identification of SM gene clusters in fungi, the region corresponds to one of five SM clusters in *B. cinerea* Chr 1, between genomic coordinates 4,002,878 and 4,093,700 (Figure 5B). To support this prediction, we analyzed the expression pattern of all genes (Figure 5C) in this putative BGC using BEB. All genes displayed highly similar expression trends across all culture conditions (Figure 5D). This pattern is consistent with transcriptional co-regulation, as expected for a SM gene cluster, most likely involved in ferrichrome siderophore biosynthesis. Since BGCs usually contain a gene encoding a TF, we searched for a regulatory protein in Region 5 (Figure 5C). Employing a manually curated catalog of TFs for *B. cinerea* [66], we identified the Bcin01g11510 gene, which encodes a fungal Zn(2)-Cys(6) binuclear TF with an unknown role in the regulation of this BGC or siderophore biosynthesis. Interestingly, this TF displays the most distinct expression pattern among all genes in the SM biosynthesis cluster, as determined using the BEB gene clustering function (left-most colored column of Figure 5D). Siderophores are complex low-molecular-weight molecules involved in the acquisition of iron, a critical micronutrient. Little is known about iron acquisition pathways and transcriptional regulatory mechanisms in *B. cinerea* [67]. However, this fungus is expected to synthesize at least nine siderophores to support metal acquisition [40,68] in addition to its membrane-bound reductive iron assimilation mechanism [41].

These findings collectively illustrate the ability of BEB to reveal the expression patterns of orphan gene clusters. This information can facilitate their investigation under specific experimental conditions. Combined with easy-to-use tools, such as antiSMASH [52], and up-to-date TF databases [66], the gene expression patterns determined using BEB could also provide a basis for the development of testable hypotheses regarding transcriptional regulation.

### 3.4. Chromosome-Wide Gene Expression Analysis

The latest iteration of the genome sequencing process of the *B. cinerea* B05.10 strain revealed exciting structural features, including two previously undetected minichromosomes [39]. In *B. cinerea*, Chr 17 and 18 comprise 18 and 16 protein-encoding genes, respectively. These accessory chromosomes (AC) are generally small and are not considered essential for survival. In some pathogenic fungi, they are characterized by the presence of genes encoding virulence factors [69,70,71]. The *B. cinerea* genes on ACs display little or no similarity to genes in other organisms, including fungi [39]. Despite the comparative analyses described in Supplementary Material 4, the vast majority are annotated as proteins with hypothetical/unknown functions. Therefore, this group of genes represents an opportunity for testing the utility of BEB by determining whether they exhibit gene expression trends that shed light on their functions.

**Figure 6:**
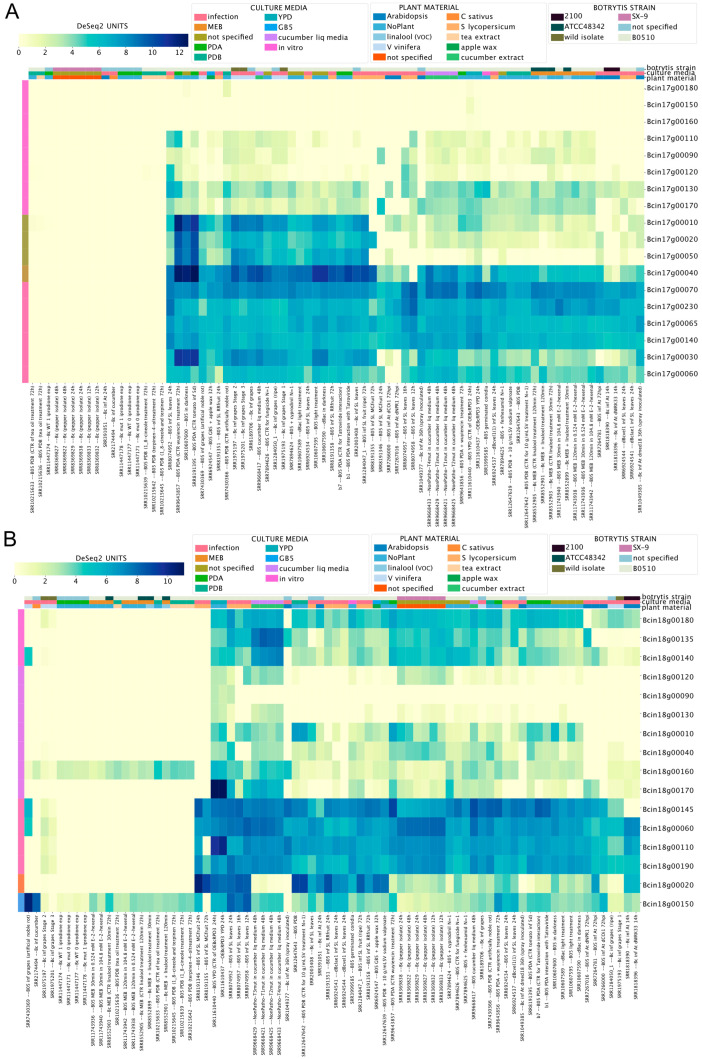
Expression levels of transcripts on mini chromosomes 17 (**A**) and 18 (**B**) of *B. cinerea*. Both heatmaps indicate mRNA levels specified by a scale from yellow to dark blue (low or high expression, respectively). Both heatmaps indicate mRNA levels and experimental conditions, as described in former figures.

Figure 6A,B show that three genes on each chromosome (Chr17: Bcin17g00150, Bcin17g00160, and Bcin17g00180; Chr18: Bcin18g00090, Bcin18g00120, and Bcin18g00130) display low mRNA levels across all RNA-Seq libraries. Interestingly, Chr17 genes Bcin17g00010, Bcin17g00020, Bcin17g00040, and Bcin17g00050 displayed the highest and most likely co-regulated gene expression, as deduced by the BEB clustering function (Figure 6A). As shown in Supplementary Material 4, the latter gene encodes a putative NRPS-like protein, whose participation in the biosynthesis of any low-molecular-weight peptidic product has not yet been described. The Chr18 genes Bcin18g00020 and Bcin18g00150 displayed distinct expression patterns during the infection of *A. thaliana* and *S. lycopersicum* (Figure 6B). While these results suggest that these genes play a role in the infection process, further experimental validation is needed. However, we cannot rule out the possibility that genes with no detectable mRNA levels are expressed in untested experimental conditions, as reported in *Aspergillus* [72].

### 3.5. Inspecting the Expression of Virulence Factors Detected in Proteomics Studies

**Figure 7:**
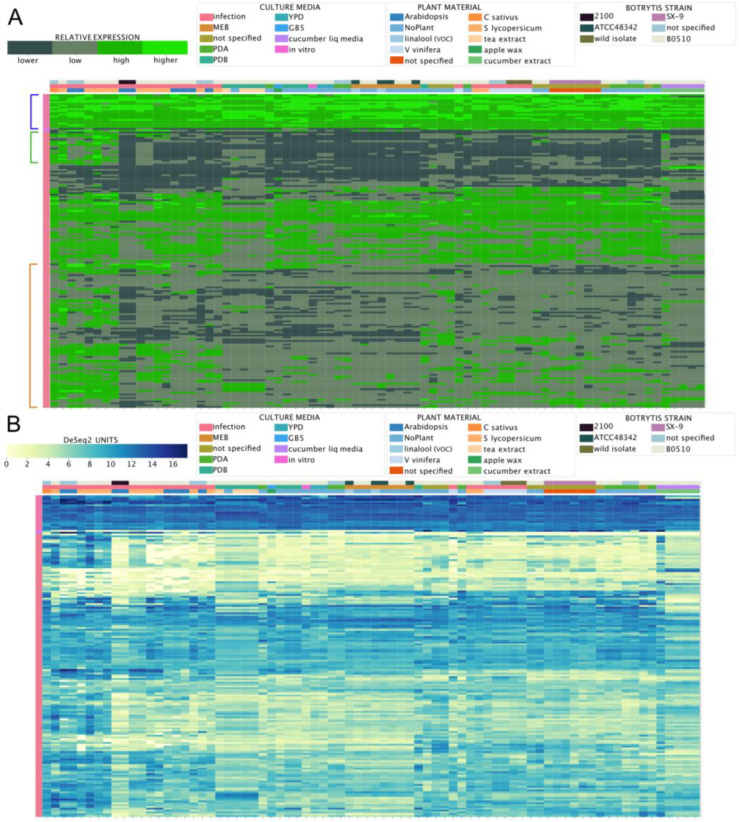
mRNA levels of virulence factors detected in proteomics studies. (**A**) A heatmap depicting the transcript levels of 176 protein-encoding genes whose products were detected in proteomics studies, as determined using the BEB quartile-categorized expression option (lower: dark green; higher: light green) or the continuous color scale (**B**) from low to high expression (yellow to dark blue). Owing to the number of genes being analyzed, neither culture conditions nor gene IDs are included in the figure (see Supplementary Material 5). In (**A**), genes with high and stable patterns of expression across conditions are indicated with a blue square bracket, while two groups of genes displaying an *in planta* “induced” pattern of expression are indicated with green and orange square brackets.

Considering the importance of virulence factors in phytopathogens, such as *B. cinerea*, we next used the BEB to analyze the expression of virulence genes, distinct from those required for phytotoxins. In general, virulence factors have a wide range of chemical properties. However, they are characterized by their ability to cause harm or to suppress/interfere with host defense strategies, leading to more damage [73]. For these reasons, elevated expression during the infection of different tissues and plant species is expected. To identify secreted virulence factors, previously published proteomic studies have taken advantage of in vitro cultures supplemented with various plant-derived components to induce their expression [74,75,76,77]. The proteins identified in these studies are summarized in Supplementary Material 5. We analyzed the expression levels of the genes encoding these proteins across experimental conditions using the BEB. As depicted in Figure 7, two different expression patterns were observed: (i) high, stable expression levels, as denoted in the upper section of the heatmap (blue square bracket), and (ii) an *in planta* “induced” pattern of expression with two groups of genes indicated by green and orange square brackets (Figure 7). In the former group, genes including several unexpected extracellular proteins were found, including *bcactA* encoding actin, *bcatp2* encoding the mitochondrial ATP synthase (beta chain), and genes encoding two additional mitochondrial proteins, malate dehydrogenase, and aconitase. Interestingly, *bcspl1* and *bcpg1*—which play significant roles in virulence—were observed in this group [78,79]. Among *in planta* “induced” genes within the green square bracket mentioned above, several glycoside hydrolases were identified (families 5 (three), 6, 7 (two), 10, 11, 28 (two), and 53).

There were also two cutinases and two pectinesterases, possibly reflecting a transcriptional regulatory mechanism involving this group of genes encoding carbohydrate-acting enzymes (Supplementary Material 5). *In planta* “induced” genes shown in an orange square bracket (Figure 7) include previously identified virulence factors, such as *bcpg3*, *4* and *6*, *bcpgx1*, *bcxyn11A*, *bcpme1* and *2*, and *bccutA*, among others. Again, while a common transcriptional regulatory mechanism is likely, little is known about the specific TFs controlling the expression of these genes.

### 3.6. Revisiting the Expression of Known Genes and Proposing New Reference Genes for Transcript-Level Analyses in B. cinerea

We integrated and analyzed data spanning a wide variety of experimental conditions (Supplementary Material 1). Thus, exploiting the wealth of data, we revisited the expression of known and previously validated reference genes in *B. cinerea* in RT-qPCR studies [80,81] and propose new candidates. By definition, reference genes tend to be constitutively expressed in all cells of an organism, with slight variation across experimental conditions. Normalized RT-qPCR experiments rely on an adequately validated (stable) reference gene(s) [82]. In addition, accurate normalization usually requires multiple validated reference genes [83], and the exact number is dependent on an experimental assessment of variability across the samples of interest [84]. Consequently, we used the BEB database to identify new reference genes, as described in the Materials and Methods. 

**Figure 8:**
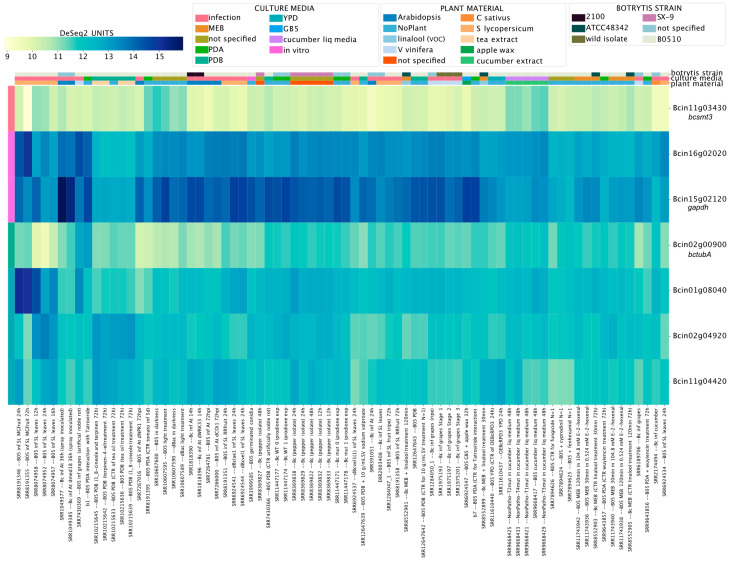
Transcript levels of previously validated reference genes employed in RT-qPCR studies of *B. cinerea*. The heatmap depicts transcript levels obtained using BEB, with a continuous color scale from yellow to dark blue indicating low or high expression, respectively. A detailed description of each experiment is provided at the bottom of the heatmap. Experimental conditions and genes are depicted in columns and rows, respectively. Gene IDs are provided at the right of the heatmap.

We first revisited the expression of seven genes used as a reference in previous RT-qPCR assays [80,81] (Supplementary Material 6). Levels of Bcin11g03430 (*bcsmt3*) and Bcin02g00900 (tubulin, *bctubA*) showed low coefficients of variation (CV) of 0.35 and 0.36, respectively, indicating little variation across all experimental conditions, as expected (Figure 8). In contrast, Bcin15g02120 (CV = 1.4), encoding the commonly used reference gene glyceraldehyde-3-phosphate dehydrogenase, displayed obvious variation in expression levels across experiments. Indeed, according to our analysis, this is the least appropriate reference gene among those used in previous studies (Supplementary Material 6 and 7). In the fungus *N. crassa*, this gene is under the control of its well-known circadian clock [85], a complex molecular machinery that in *B. cinerea* modulates time-dependent fungal–plant [86] and fungal–fungal dynamics [87].

To provide an additional tool for *B. cinerea* research, we also generated a list of 40 new reference genes showing the lowest CVs across conditions in each global gene expression quartile. Importantly, the proposed reference genes need to be further validated by RT-qPCR, i.e., for intermediate-low and intermediate-high expression levels (second and third quartile of normalized expression, respectively) (Supplementary Material 7). Nevertheless, as shown in Figure S2, their expression levels were highly similar in different experimental conditions. 

## 4. Conclusions

Based on carefully curated RNA-Seq datasets for *B. cinerea*, the first implementation of the BEB was developed. This tool lets users directly explore global gene expression trends in this fungus using a simple yet powerful online graphical user interface. An important feature of BEB is its visualization options, including the clustering of gene expression values. As demonstrated in Figure 5, BEB can be used for the identification of a cluster of co-regulated genes, delineating a testable hypothesis on gene function based entirely on gene expression. Broadly speaking, we hypothesized that cross-laboratory RNA-Seq analyses of *B. cinerea* can reveal “hidden” global gene expression trends that can be exploited to contribute to our understanding of gene function. Notably, the modularity of BEB and its simple installation procedure in Linux-based operative systems could facilitate its adoption in other (fungal) organisms, expanding the potential impact of the tool. As more publicly available data become accessible, we will extend the BEB database to keep it up-to-date. Importantly, this can be done expeditiously as it is only necessary to re-run the described procedure on the experiments lacking from the database to rapidly append the results to the information on the BEB server. 

## Figures and Tables

**Table 1:** Overview of RNA-Seq experiments and data in the NCBI Short Read Archive (SRA ^1^) for the 10 most relevant fungal phytopathogens.

Fungal Species	SRA Experiments	SRA Studies
*Magnaporthe oryzae*	1714	125
*Botrytis cinerea*	2403	89
*Puccinia* spp.	3847	195
*Fusarium graminearum*	2141	177
*Fusarium oxysporum*	2700	188
*Blumeria graminis*	1057	43
*Mycosphaerella graminicola*	2095	230
*Colletotrichum* spp.	1752	219
*Ustilago maydis*	538	38
*Melampsora lini*	205	3

^1^ Data were retrieved from https://trace.ncbi.nlm.nih.gov/Traces/index.html?view=study (accessed on 30 November 2021).

## Data Availability

The data and code supporting the reported results can be found in the following repository (https://github.com/bioquimico/biber/tree/main/biber).

## Supplementary Materials

The following supporting information can be downloaded at: https://www.mdpi.com/article/10.3390/jof9010084/s1, Figure S1: BEB data processing flow chart; Figure S2: mRNA levels of proposed reference genes that can be employed in future RT-qPCR experiments after proper validation; Supplementary Material 1: BEB’s metadata file; Supplementary Material 2: Gene IDs and names of the two major phytotoxic secondary metabolites of *B. cinerea*; Supplementary Material 3: Gene IDs and names of key enzymes required for the synthesis of secondary metabolites (SM) in *B. cinerea* as described previously [65,88,89,90,91]; Supplementary Material 4: Functional annotation of mini-chromosomes (Chr 17 and 18) with protein-encoding genes. The data were obtained after BLAST2GO analysis; Supplementary Material 5: Previously identified extracellular *B. cinerea* proteins detected in *in vitro* cultures supplemented with plant-derived material. The colors of the groups refer to those in Figure S1. Supplementary Material 6: Reference genes validated as such in *B. cinerea* RT-qPCR assays. The rank number was calculated based on the data presented in Supplementary Material 7; Supplementary Material 7: Proposed reference genes for RT-qPCR studies. The table shows each gene (ten for each expression quartile), as well as its coefficient of variation (CV).
