# Supplementary figures and images for: The Botrytis cinerea Gene Expression Browser
Source: J Fungi (Basel). 2023 Jan 6;9(1):84. doi: 10.3390/jof9010084 (PMC9861337; doi:10.3390/jof9010084)

## Botrytis cinerea Expression Browser (BEB) Data Processing Flow Chart

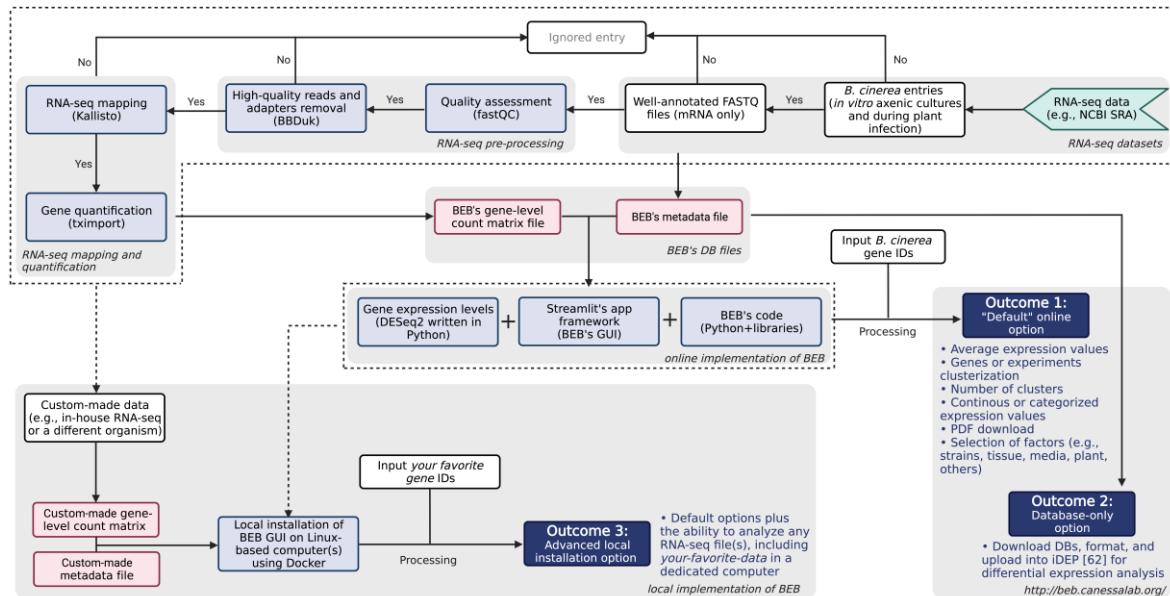

Supplement: Supplementary file 1 [file jof-09-00084-s001.zip › Supplementary Figure S1 New.pdf]

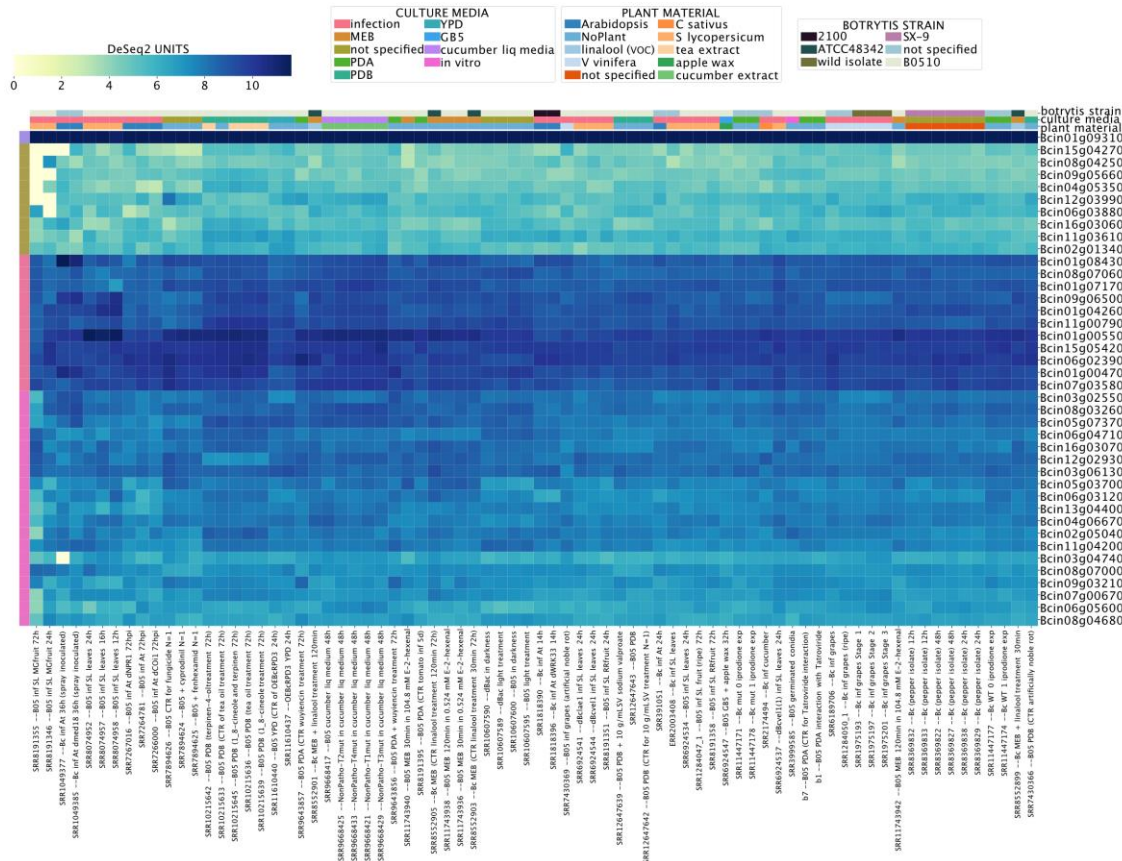

Supplement: Supplementary file 1 [file jof-09-00084-s001.zip › Supplementary Figure S2.pdf]
